# Consistency Between Administrative Health Records and Self-Reported Health Status and Health Care Use Among Indigenous Wayuu Health Insurance Enrollees: La Guajira, Colombia

**DOI:** 10.1177/01632787241263370

**Published:** 2024-06-17

**Authors:** Aynslie Hinds, Beda Suárez Aguilar, Yercine Duarte Berrio, Dorian Ospina Galeano, John Harold Gómez Vargas, Valentina Espinosa Ruiz, Javier Mignone

**Affiliations:** 1 8665 University of Winnipeg, Canada; 2 Anas Wayuu, Colombia; 3 8664 University of Manitoba, Canada; 4 27983 Universidad de Antioquia, Colombia

**Keywords:** survey/administrative data, Indigenous health data, consistency self-reported and administrative data, Indigenous health, Colombia, Wayuu

## Abstract

The objective of the study was to assess the consistency between self-reported demographic characteristics, health conditions, and healthcare use, and administrative healthcare records, in a sample of enrollees of an Indigenous health organization in Colombia. We conducted a phone survey of a random sample of 2113 enrollees September-2020/February-2021. Administrative health records were obtained for the sample. Using ICD-10 diagnostic codes, we identified individuals who had healthcare visits for diabetes, hypertension, and/or pregnancy. Using unique identifiers, we linked their survey data to the administrative dataset. Agreement percentages and Cohen’s Kappa coefficients were calculated. Logistic regressions were performed for each health condition/state. Results showed high degree of agreement between data sources for sex and age, similar rates for diabetes and hypertension, 10% variation for pregnancy. Kappa statistics were in the moderate range. Age was significantly associated with agreement between data sources. Sex, language, and self-rated health were significant for diabetes. This is the first study with data from an Indigenous population assessing the consistency between self-reported data and administrative health records. Survey and administrative data produced similar results, suggesting that Anas Wauu can be confident in using their data for planning and research purposes, as part of the movement toward data sovereignty.

## Introduction

Broadly speaking, the two main types of data sources in population health and health services research are surveys and administrative/clinical records. As is the case with other fields of research, the main challenge is to ensure the reliability and validity of the data. Since earlier years of health and sociological research, methodological studies of survey research have dealt “with the many possible sources of bias to be found in… surveys” (Suchman, 1962). Furthermore, given that self-reporting “is often used to estimate health care utilization … the accuracy of such data is of paramount concern” (Bhandari & Wagner, 2006). Studying the consistency between self-reported and administrative health records is of value for health services research, due to their respective strengths and weaknesses, as well as advantages and disadvantages. It has been suggested that linking administrative data with primary data complements the unique strengths of each type of data (Roos et al., 1993). Furthermore, the consistency between self-reported and administrative health records may substantially vary across different types of illnesses, population groups, contexts, and countries. Thus, a better understanding of their levels of trustworthiness can provide evidence for methodological decision-making in health research. To our knowledge, despite the numerous studies assessing this consistency (Al-Azazi et al., 2019; Brown & Adams, 1992; Clifasefi et al., 2011; Lix et al., 2008; Lix et al., 2010; Okura et al., 2004; Rhodes & Fung, 2004; Robinson et al., 1997), none has been conducted with Indigenous populations. In a two-year study with a large Indigenous health organization in Colombia, we assessed the role of intercultural hostels and bilingual guides in relation to access to health care, utilizing self-reported survey data and administrative data (Mignone et al., 2021). This provided a unique opportunity to assess the consistency between administrative health records and self-reported data from the Indigenous Wayuu population of Colombia.

The Wayuu are the largest of the 115 Indigenous ethnic groups in Colombia (Departamento Administrativo Nacional de Estadística, 2019). Living in the northeast region of Colombia, La Guajira, in 2018 the Wayuu population consisted of 380,460 people (Departamento Administrativo Nacional de Estadística, 2019). For livelihood, the Wayuu raise small livestock, produce, and sell hand-woven items such as hammocks and handbags, aside from relying on other informal income sources. A majority of the Wayuu live in small rural villages and hamlets, some closer to urban centres and some in remote areas (Mignone et al., 2021). From an epidemiological perspective, frequent pathologies include malnutrition, respiratory and gastrointestinal infections among children less than five years of age (Hernández Bello et al., 2017), sexually transmitted infections, hypertension, uterine/cervical cancer, injuries, and dental problems (Mignone et al., 2021).

In 1993, the Colombian government passed Law 100 creating Health Promoting Enterprises (Empresas Promotoras de Salud [EPS]) (Restrepo & Valencia, 2002), which in essence are health insurance organizations. Some EPSs are for-profit and others not-for-profit. Anas Wayuu is a non-profit Indigenous led EPS created in early 2000 by two Indigenous associations representing 120 Wayuu communities. At the time of our study, Anas Wayuu was providing health care coverage to 220,000 people, 71% Indigenous Wayuu. To provide coverage, Anas Wayuu contracts with a health care network of 27 Indigenous Health Service Provider Institutions and 65 non-Indigenous Health Service Provider Institutions privately owned or from the public sector such as State Social Enterprises. The coverage includes health promotion and disease prevention services, and primary, secondary, and tertiary health care (Mignone & Gómez Vargas, 2015). As the single payer of health services, Anas Wayuu relies on a vast information system of administrative data for all health care encounters.

For the main study (assessing Anas Wayuu’s intercultural health initiatives and their role in access to health care) (Mignone et al., 2021), we utilized data from the administrative health records information system, as well as survey data. The purpose of the present study was to examine the consistency between survey responses and administrative health records among Indigenous Wayuu health insurance enrollees in relation to health conditions/states and use of healthcare services, and to determine the factors associated with the consistency of responses.

## Method

### Cohort

The sampling frame, consisting of 34,961 Wayuu individuals 18 years of age and older, was created from Anas Wayuu’s lists of current (90%) and former (10%) enrollees. During the data collection phase of the study (September 2020 to February 2021), four bilingual surveyors attempted to contact 25,025 individuals by phone. Of those, 22,719 were unreachable (phones turned off, numbers changed, etc.), and 371 declined to participate. A total of 2160 surveys were completed. After removing duplicates and poorly completed surveys, the final sample consisted of 2113 individuals.

### Study Period

The study period was 2017 to 2020. We chose this period because it closely aligned to the three years prior to the survey, which was the timeframe that the survey questionnaire asked about.

### Data Sources

The 34-item survey was jointly developed among researchers, Anas Wayuu staff, and a Wayuu Knowledge Keeper. The questions asked about demographic characteristics (e.g., sex, age, region of residence), languages spoken (Spanish, Wayuunaiki), self-rated health (poor to excellent), diagnosed physical health conditions, and receipt of healthcare in the past three years. Sex was dichotomous male/female as per advice of Anas Wayuu, which also matches the two categories used for administrative data. The survey was translated from Spanish to Wayuunaiki so it could be administered in the participants’ preferred language.

The study used administrative data collected by Anas Wayuu which in Colombia are called “Registros Individuales de Prestación de Servicios” (RIPS) [Individual Records of Service Provision]. The RIPS data contains information on aspects of medical service delivery, such as hospital discharge summaries, emergency visits, medical claims, prescription drug therapies, and birth registrations. The RIPS also contain demographic and identifying information, such as age, gender, area of residence, and user type. The RIPS structure has been unified and standardized for all health institutions in the country. Health institutions must provide monthly health records to a regional state entity. According to an official data quality report, when comparing the RIPS to patients’ clinical history, there was 95% agreement between the data sources for patient demographic characteristics and 83.4% agreement for diagnostic coding (Martínez & Pacheco, 2013).

As an entity that manages public resources, Anas Wayuu relies on this healthcare data to pay healthcare providers and to manage resources. Additionally, RIPS are the primary data source used to estimate the prevalence and incidence of diseases among the enrolled population (Martínez & Pacheco, 2013). Although administrative data is not intended for research purposes, it is a rich source of information for studying enrollee utilization of healthcare resources, as well as other evaluation and research purposes (MCHP, 2007).

### Variables

The survey demographic question included sex, languages spoken, and region of residence. Age was determined as the difference between the interview date and birthdate recorded in the information system of Anas Wayuu enrollees. The languages question asked if participants could “entiendo” (understand) and/or “hablo” (speak) Castellano (Spanish) and/or Wayuunaiki. The self-rated health question was worded as follows: “¿En general, diría que su salud es?” (In general, would you say your health is?). Respondents could answer “excelente”, “muy buena”, “buena”, “regular”, or “pobre” (excellent, very good, good, regular, poor).

The health conditions question in the questionnaire asked about ever having cardiovascular diseases, respiratory diseases, stomach issues, cancer, diabetes, infectious diseases, and being pregnant. We focused on three conditions/states: hypertension, diabetes, and pregnancy. We selected these three conditions/states because we hypothesized that individuals would seek healthcare for these conditions/states. In fact, hypertensive diseases are one of the leading causes of medical consultation in La Guajira (Ministerio de Salud de Colombia, 2012). We also hypothesized that since hypertension and diabetes require treatment and monitoring, people would know if they had these conditions and would accurately self-report them. We also figured that women would accurately self-report pregnancy. We hypothesized that the degree of agreement would vary across the health conditions. We did not choose cancer because too few survey participants self-reported having a cancer diagnosis. We did not select respiratory diseases, stomach issues, or infectious diseases because they are general categories of conditions that people may not seek healthcare for. The survey question about healthcare use asked about using or trying to use health services (e.g., doctor, nurse, treatment) in the past three years. Individuals responded either “Si” or “No” (yes or no) to the health conditions and healthcare use questions.

Enrollment records were linked to RIPS to consolidate patient history of use of health services and clinical and demographic information. The four-digit ICD-10 disease diagnosis code and date of visit were used to identify patients with diabetes, hypertension, and those who were pregnant during the study period. “Cases” were defined as survey participants with at least one visit to the doctor or hospital with the ICD-10 diagnostic code for the health condition/state. The ICD-10 codes were chosen based on a review of how other studies defined these conditions/states using administrative data (Chen et al., 2010; Khokhar et al., 2016; Robinson et al., 1997).

### Data Linkage

Anas Wayuu provided a file of medical visits of 1,442,399 consultation records and 70,973 hospitalizations for years 2017 to 2020. The administrative data and survey data files were linked via a unique user identifier. Thus, the joined dataset only included records for the 2113 survey participants. The medical visits and hospitalization data were structured in a way that the number of visits per patient, diagnoses, and year could be determined. Survey participants who were in the Anas Wayuu user registry, but did not have any healthcare visits, were assumed to not have used health services during the study period.

### Statistical Analysis

We calculated basic descriptive statistics (i.e., means, standard deviations, frequencies, percentages) for the demographic variables (e.g., age, sex), health conditions/states, and the pattern of contact with the health care system (e.g., number of hospital admissions and physician visits). The prevalence of the health conditions/states was calculated from each data source. The degree of agreement between the two data sources was determined by calculating the percent agreement, and the Cohen’s Kappa coefficient (a chance-corrected measure of agreement) and corresponding 95% confidence intervals. According to Landis and Koch (1977), a kappa value less than 0.40 is considered poor-to-fair agreement, a kappa value between 0.41 and 0.60 is considered moderate agreement, a kappa value of 0.61 and 0.80 is considered substantial agreement, and a kappa value of 0.81 and 1.00 is considered excellent agreement. Logistic regression was performed for each health condition/state to determine if any of the demographic factors could explain the (in)consistency between the data sources.

## Results

### Consistency Between Self-Reported Demographic Characteristics and Administrative Records

The mean age of the sample was 39.6 years (SD = 15.9), 64.7% were women, 56.5% resided in rural areas, 77.8% spoke Spanish, 62.1% spoke Wayuunaiki, and 86.1% were current Anas Wayuu enrollees. The two data sources had virtually identical sex and age distributions (Table 1). The percentage agreement between the data sources for both gender and age group was 97.7% and the kappa was in the excellent range (Table 2). For gender, the discordance was evenly split between female-male and male-female (Table 2). For age group, the percentage of disagreement was slightly higher where ages in the administrative data were younger than the self-reported ages (33, 1.6%) (i.e., below the diagonal in Table 2) than where the self-reported ages were younger than the ages in the administrative data (15, 0.7%) (i.e., above the diagonal in Table 2).

**Table 1.** Sex and Age Distributions

Characteristic	Response	Survey		Administrative data	
		*N*	%	*N*	%
Sex	Female	1368	64.7	1370	64.8
	Male	745	35.3	743	35.2
Age	<29 years	743	35.2	759	35.9
	30 – 44 years	640	30.3	631	29.9
	45 – 64 years	544	25.7	538	25.5
	65+ years	186	8.8	185	8.8

**Table 2.** Concordance and Discordance Between the Data Sources on Gender and Age

Survey	Administrative data				Kappa (95% CI)	Sig
Gender	Female		Male			
Female	1345 (63.7%)		23 (1.1%)		0.95 (0.94, 0.96)	<0.001
Male	25 (1.2%)		720 (34.1%)			
Age groups	<29 years	30 – 44 years	45 – 64 years	65+ years		
<29 years	731 (34.6%)	6 (0.3%)	5 (0.2%)	1 (0.0%)	0.97 (0.96,0.98)	<0.001
30 – 44 years	17 (0.8%)	620 (29.3%)	3 (0.1%)	0 (0.0)		
45 – 64 years	10 (0.5%)	4 (0.2%)	530 (25.1%)	0 (0.0)		
65+ years	1 (0.0%)	1 (0.0%)	0 (0.0%)	184 (8.7%)		

### Consistency Between Self-Reported Healthcare Use and Administrative Records

Based on the administrative data, 88.1% of the sample (1861 individuals) used healthcare in years 2017 to 2020, while 84.3% of the sample self-reported they tried or used *Arijuna* (allopathic non-Indigenous) health services. Overall, 79.0% of the records agreed (see total row in Table 3). The disagreement was higher where respondents said they did not receive healthcare, but the administrative records suggested otherwise (No-Yes) than the reverse (Yes-No). The kappa value was in the poor range (0.12). As noted earlier, 86.1% of the sample (1819 individuals) were enrolled with Anas Wayuu at the time of the survey and 13.9% of the sample (294 individuals) were not enrolled with Anas Wayuu. Since administrative healthcare records are based on enrollment status, we examined the consistency between the two data sources stratified by enrollment status. For current enrollees there was 84.3% agreement and for past enrollees there was 46.3% agreement. The kappa values for both strata were in the poor range (Table 3).

**Table 3.** Concordance and Discordance Between the Data Sources with Respect to Healthcare Use

Analysis	Survey-admin				Kappa (95% CI)	Sig
	No-No	No-yes	Yes-No	Yes-yes		
Total (*N* = 2113)	70 (3.3%)	262 (12.4%)	182 (8.6%)	1599 (75.7%)	0.120 (0.07,0.17)	<0.001
Current AW enrollees (*N* = 1819)	25 (1.4%)	236 (13.0%)	50 (2.7%)	1508 (82.9%)	0.091 (0.04,0.14)	<0.001
Past AW enrollees (*N* = 294)	45 (15.3%)	26 (8.8%)	132 (44.9%)	91 (31.0%)	0.028 (−0.06,0.11)	0.530

### Consistency Between Self-Reported Health Conditions and Administrative Records

The prevalence of diabetes and hypertension was almost identical between the two data sources (Table 4), while there was a ∼10% difference in the percentage who were pregnant between the two data sources. The pregnancy analysis was limited to respondents who were female and less than 50 years old (using sex and age from the survey).

**Table 4.** Prevalence of the Health Conditions/States by Data Source (*N* = 2113)

Condition	Survey		Administrative data	
	Yes	No	Yes	No
Diabetes	7.5	92.5	7.9	92.1
Hypertension	14.6	85.4	14.7	85.3
Pregnancy (*N* = 1050)	34.0	66.0	43.8	56.2

To have an ICD-10 code for hypertension, diabetes, and pregnancy, participants must have accessed healthcare. Thus, to examine the consistency between the two data sources for the health conditions/states, we limited the sample to only current Anas Wayuu enrollees who used healthcare (Table 5). For diabetes, 91.5% of the records agreed, resulting in a kappa in the moderate range. For hypertension, 84.5% of the records agreed, also resulting in a kappa in the moderate range. The pregnancy analysis showed a degree of agreement between the two data sources of 73.3%; the kappa was in the moderate range. The way the two data sources were discordant (i.e., No-Yes, Yes-No) was similar for hypertension and diabetes. For pregnancy, the disagreement was three times higher for participants who self-reported they were not pregnant, but the administrative data suggested otherwise (No-Yes) than the reverse (Yes-No).

**Table 5.** Concordance and Discordance Between the Two Data Sources for the Health Conditions/States

Health condition	Survey-admin				Kappa (95% CI)	Sig
	No-No	No-yes	Yes-No	Yes-yes		
Diabetes (*N* = 1744)	1512 (86.7%)	81 (4.6%)	67 (3.8%)	84 (4.8%)	0.49 (0.41, 0.56)	<0.001
Hypertension (*N* = 1744)	1315 (75.4%)	151 (8.7%)	119 (6.8%)	159 (9.1%)	0.45 (0.39, 0.50)	<0.001
Pregnancy (*N* = 902)	404 (44.8%)	185 (20.5%)	56 (6.2%)	257 (28.5%)	0.46 (0.41, 0.52)	<0.001

### Logistic Regression Results

Logistic regression was performed to determine if agreement between the two data sources for the three health conditions/states could be explained by the demographic characteristics (sex, age, language) and health status. These demographic and self-rated health variables were obtained from the survey. The sample was limited to survey participants who were current enrollees at the time of the survey and used healthcare in the three years prior to their survey date. The Hosmer-Lemeshow test was not significant for any of the models suggesting the models fit the data well (diabetes: χ_(8)_^2^ = 13.292, *p* = .102; hypertension: χ_(8)_^2^ = 8.882, *p* = .180; pregnancy: χ_(6)_^2^ = 3.622, *p* = .728). The Nagelkerke *R*^2^ was 0.07 for the diabetes model, 0.095 for the hypertension model, and 0.16 for the pregnancy model, suggesting the models did not explain much of the variation.

Age was significantly associated with agreement for all three health conditions/states. For diabetes and hypertension, the odds of agreement were lower for the two middle age groups (30–64 years) than the oldest age group (65+ years). Language was only significantly associated with agreement for diabetes; specifically, individuals who spoke Wayuunaiki had lower odds of agreement between the data sources than individuals who did not speak Wayuunaiki. Sex and self-rated health were significantly associated with agreement between the data sources for diabetes. That is, male sex and having better health had lower odds of the data sources agreeing relative to female sex and having poorer health, respectively. Age was the only variable significantly associated with the data sources agreeing for pregnancy; the odds that the two data sources agreed was significantly higher for 30- to 44-year-olds relative to 45- to 49-year-olds.

## Discussion

A primary concern when using administrative data for research is the accuracy and completeness of information (Peabody et al., 2004), while recall bias is a key concern for self-reported data (Lix et al., 2010). Given that the consistency between self-reported and administrative health records is likely to vary across different population groups, contexts and countries, the lack of such studies among Indigenous populations is a significant gap in the literature. The data from our broader study (Mignone et al., 2021) provided us with the opportunity to examine the consistency between survey responses and administrative health records among Indigenous Wayuu health insurance enrollees. This assessment was done in relation to demographic information, health conditions/states, and use of healthcare services, while seeking to determine the factors associated with consistent responses.

The sex and age distributions derived from the survey and administrative data were almost identical and there was a high degree of agreement between them, suggesting both data sources are accurate and reliable with respect to these characteristics. This was expected since sex and age are unambiguous. A similar percentage of survey respondents self-reported using or trying to use healthcare in the three years prior to the survey (84%) as actually used healthcare according to Anas Wayuu’s records (88%); however, the kappa statistic was in the poor range, because the observed agreement (79%) was similar to the chance agreement (76%). As well, the kappa statistic is influenced by prevalence; that is, the level of agreement will be lower when the prevalence is high (in this case, a high percentage of the sample used health care) (Zec et al., 2017). The discrepancy between the data sources may be partly due to how the survey question was asked (i.e., used or *tried to* use healthcare); however, we would have expected the percentage of healthcare users to be higher based on the survey than the administrative data. The agreement between the data sources in relation to healthcare utilization (88% for administrative and 84% for survey) seems to conform with the results from other studies (Short et al., 2009) and somewhat differ from others (Beebe et al., 2006). Thus, at a population-level, Anas Wayuu can be relatively confident in reporting on the healthcare use of its enrollees.

The prevalence of diabetes and hypertension were almost identical between the data sources; however, there was only a moderate degree of agreement. Interestingly, agreement between the data sources was significantly associated with some of the characteristics of the sample, meaning that agreement was better for certain subgroups than others. Specifically, there tended to be better agreement for women, older adults, individuals who did not speak Wayuunaiki, and individuals in poorer health.

There was approximately a 10% difference among women of reproductive age who self-reported being pregnant in the three years prior to the survey (34%) and the administrative case definition (44%), and the kappa statistic was in the moderate range. Our administrative case definition may have been too sensitive, “detecting” cases of pregnancy when a woman had not been pregnant (e.g., we included ICD-10 code Z40.0 -problems related to an unwanted pregnancy-) or respondents may have answered the question negatively if their pregnancy did not result in a live birth. Some of the misclassification may have also occurred due to inaccuracies in sex and age (the variables used to subset the sample), though this misclassification is likely minimal. In contrast to the diabetes and hypertension results, agreement between the data sources tended to be better for younger women and women in better health. Our findings in general are consistent with factors that affect the accuracy of self-reporting of health care utilization identified in a systematic review of the literature: sample population and cognitive abilities; recall time frame; type of utilization; utilization frequency; questionnaire design; mode of data collection; and memory aids and probes (Bhandari & Wagner, 2006).

There were limitations to the study. The survey was not conducted face-to-face but over cell phones. Furthermore, it was administered during a time of uncertainty such as the COVID pandemic. It is difficult to ascertain if the results would have been different if the survey had been conducted before or after the pandemic.

## Conclusion

At a population-level, our study showed that survey and administrative data tell essentially the same story, suggesting that Anas Wayuu can be relatively confident in using their administrative data for planning and research purposes with respect to these health conditions. However, subgroup analyses based on identity characteristics, particularly age, should be interpreted with caution. The study addressed a significant gap in the literature, as it relates to health care utilization data of Indigenous peoples. Given the increasing relevance of addressing the rights of Indigenous Peoples across the world to strengthen and implement their data sovereignty (Mashford-Pringle et al., 2019; Hayward et al., 2021; Walter & Suina, 2019), the research reported here is also of value in supporting that agenda.
